# Living-Donor Liver Transplant in Oman: A Quantitative Cross-Sectional Study of Donors' Experiences and Challenges

**DOI:** 10.1155/2021/4251814

**Published:** 2021-11-15

**Authors:** Mudhar Al Adawi, Hasina Al Harthi, Raja Al Hinai, Suad Al Haddabi, Iqbal Al Busaidi, Omar Al Siyabi, Salah Thabit Al Awaidy

**Affiliations:** ^1^Department of Nursing, Royal Hospital, Ministry of Health (MOH), Muscat, Oman; ^2^Department of Training and Studies, Royal Hospital, Muscat, Oman; ^3^Divisions of Gastroenterology and Hepatology, Department of Medicine, Royal Hospital, Muscat, Oman; ^4^Office of Health Affairs, Ministry of Health, Muscat, Oman

## Abstract

**Background:**

In Oman, the first liver transplant was performed at the Royal Hospital (RH) in September 2017. Since then, thirteen cases have been operated on at the RH. All of these cases were living-donor liver transplants (LDLT), and the remaining cases were treated in India with a total of approximately 193 recipients. To provide an in-depth overview of donor experiences, challenges, and perceptions, a cross-sectional study was conducted.

**Methods:**

A cross-sectional study was conducted at one tertiary hospital in 2019. The survey was designed to collect data composed of closed and open-ended questions to reveal a thorough knowledge of the topic.

**Results:**

A total of 50 of 120 donors responded to the survey with male dominance in the sample (68%) and 64% were aged 28 to 38 years. 66% of the respondents came to know about the donation through hospital staff. Interestingly, respondents (*n* = 8/12) who reported that fear of operation is the cause that prevents people from donating are among the male gender, while more men believe that the main cause is lack of knowledge. 90% of the respondents felt satisfied after donation. More men reported ambiguous feelings before donation. Moreover, married donors reported ambiguous feelings before donation (*p* = 0.008). The younger age group reported anxiety and doubt as a challenge through their donation experience.

**Conclusion:**

This study revealed that donors have a positive feeling after donating as they have saved a life, as well as being empowered by family and community. The donors encourage individuals to donate a portion of their liver. Some crucial questions arose, such as anxiety before surgery, ambiguous feelings before surgery, and fatigue after surgery. These findings underscore the importance of a holistic approach that would enable donors to be well informed prior to surgery.

## 1. Introduction

Since liver transplant was first performed by Thomas Starlz in 1963 [1], it has become a life-saving treatment option for patients suffering from terminal liver disease [2]. Living-donor liver transplantation (LDLT) is an option in places where deceased-donor liver is not readily available, such as in Oman. The willingness of donors to donate part of their liver to a parent or friend is strongly influenced by the social, religious, and educational level [3].

Donating a portion of one's liver to a family member or close friend is a medical “hero” for the recipient and for the community at large [4]. Ensuring donors' safety and prevention of surgical failure is an absolute priority. Therefore, potential donors are subject to a multiphase evaluation protocol to avoid any situation that could increase operational risks.

Recent research in New York by Rudow D et al. [5] showed that, of the 220 living donors, the majority reported high rates of willingness to redonate (>90% in the fifth year after donation), feeling “very satisfied” after donating (81% to 88% over time), and greater positive perspectives and self-esteem in liver transplantation (82%).

In Oman, the first hepatic transplant was carried out at the Royal Hospital (RH) on September 24, 2017.

Since then, 13 patients were operated on at the RH which is all LDLT. All of these cases were LDLT, and the remainder were treated in India with a total of approximately 193. Recipients and donors are initially assessed as part of our national hospital transplantation program. A thorough informed consent process and rigorous medical assessments of donors and recipients are conducted to ensure proper ethical practice and safety.

Once deemed appropriate for LDLT, a medical report is sent to the transplant team in India, who will review all the details of the reports and render the final decision for the operation. All individuals who have undergone surgery (donors and recipients) are tracked at RH.

In March 2021, the Oman National Transplant Programme for Tissues and Organs was established to organize, develop, and plan the transplant process in Oman. To enhance the donation process in Oman and to obtain detailed information on donor experiences, challenges, and perceptions, a cross-sectional study was performed and discussed in this study.

## 2. Methods

### 2.1. Study Setting

The study was conducted at a tertiary hospital in Oman which performs liver transplants in the country.

### 2.2. Study Design and Population

This cross-sectional study was carried out from November 2019 to January 2020. Since the beginning of liver transplantation in Oman, the total number of donors is 193 for citizens and noncitizens, where some donors are deceased, which is not directly related to the donation. Therefore, the sample size reflected the fact that the total population met the inclusion criteria. Noncitizens or cadaver donors were excluded.

### 2.3. Study Tool

The survey was designed in Arabic as the target participants are Arabic speakers. The questionnaire consisted of closed and open-ended questions to reveal an in-depth understanding of the subject. A total of 35 open and closed questions were structured, prompting donors to talk freely and reflect on their experience of donating liver. The survey tool consisted of the following elements: previous knowledge of LDLT; challenges faced by donors and how to overcome them; willingness to donate following death; the justification for LDLT; the psychological and social status of donors after donation; the impact of the donation on the nature of the donors work; acceptable levels of risk; possible donor complications; and support for LDLT from liver transplant team.

This survey was commissioned by two field experts who are liver specialists and a research expert from the WHO office in Oman to verify the validity of the content. As the target population is Arabic-speaking, the tool was translated into Arabic by all researchers and then handed over to a native Arabic speaker to verify the translation.

The survey was piloted with five donors for validation, which was then generated as an online questionnaire.

### 2.4. Data Collection

The online link to the survey was distributed by liver transplantation nurses using the clinic's contact list of liver donors. All donors enrolled in the clinic were provided with the online link and were invited to participate in the study. A one-month period was allotted for complete data collection, and a weekly reminder was sent to all donors via a liver transplant nurse.

### 2.5. Statistical Analysis

Descriptive analysis was done to describe the characteristics of the participants such as percentage, frequency, counts of the gender, level of education, and source of income as shown in Table 1. Chi-square analysis was conducted to correlate demographic details (gender, age, and education level) with the three domains of the tool (donation process, preparation, support, and challenges).

### 2.6. Ethical Consideration

This study was approved by the Royal Hospital Scientific Research Board under approval number 94/2019. Participants in this study were informed of the objective of the research, and voluntary participation was explained via an online link prior to the questions. In addition, participants were informed through the link that once they clicked on the bottom of the agreement, it is an agreement to participate in the study. In addition, no details were required for the tool for identifying the specific participant. To secure the collected data, all data were transmitted online to the primary author.

## 3. Results

### 3.1. Demographic Characteristics

In total, 50 donors out of 120 have participated in this survey. Males dominated the sample (38; 68%), and 32 (64%) were between the ages of 28 and 38, with no significant gender differences observed. 26 (52%) had a secondary or higher level education, 20 (40%) were unemployed, and the majority of respondents (donors) were the recipient's parent (either mother or father of the recipient) (Table 1).

### 3.2. Donation Process

Two-thirds of the respondents became aware of the donation through hospital staff, with no significant difference between gender, age group, or relationship to the patient (Table 2). The majority of the respondents (28; 56%) are aware that 60–65% of their liver is taken to the donation with no significant difference in relation to their educational level (*p* = 0.758). However, there is a significant relation to age group (*p* value = 0.006) in terms of awareness of liver donation size.

Interestingly, respondents (*n* = 8/12) who reported that fear of operation is the cause that prevents people from donation are among male gender, while more men believe that the main cause is lack of knowledge. The majority of men and older groups disagree that postdonation body image may affect the donor's decision to give. Older groups supported organ donation after death, while younger groups were neutral, with no significant difference in educational attainment (*p* = 0.084). However, all of them disagreed with higher education.

### 3.3. Preparation of Donors

Overall, 41 (82%) of respondents agreed that they had prepared the donation transaction well before the donation. However, younger age groups reported better preparation and explanation of potential complications. Men and older adults reported improved preparedness for postoperative pain (Table 3).

### 3.4. Support of Donors

In general, most (39.78%) of the respondents reported that they were well supported by family, friends, and health care providers, although some of the younger age groups were neutral in terms of support for health care providers after donation as well as support for family and friends before donation (Table 4).

### 3.5. Donors' Experience on Donation Process

The majority of respondents (50; 90%) felt satisfied after giving, with the exception of five donors in the youngest age group with different levels of education; however, all those with secondary education were in agreement (*p* = 0.009) (Table 5). Few donors in the older age group (50; 4%) rated their donation experience as below expectations and they feel that the recipient's situation is also lower than their expectations. Both respondents belonged to the men population.

### 3.6. Challenges

In addition, more men reported an ambiguous feeling before donation, and married donors also reported an ambiguous feeling before donation (*p* = 0.008) with no significant difference calculated in relation to the education level (*p* = 0.153) (Table 6).

## 4. Discussion

Our study confirmed that liver donation is a good experience for the majority of people. In particular, self-esteem, family and community recognition of generous giving, and the sense of pride in helping a loved one have increased. Furthermore, the study participants were positive for organ donation as a result of death. These findings are documented as the donation is considered to be a positive experience [5, 6].

Based on our study, it is obvious that the majority of liver donors were primarily informed of the donation through the donation clinic and popular media such as Twitter® and whatsApp®. These results are inconsistent with [7, 8] that the transplant team was most often identified as the primary source of live donor information. This could be due to relative concerns when they have unwell family member and they seek mainly their help from trusted sources such as hospital staff or those who came through the same experience such as use of Twitter or text messages for previous donors. Moreover, participants of this study expressed they believe based on opinion that lack of knowledge could be the cause of hesitance in organ donation. This finding supports a previous study [1] that lack of knowledge is the cause of low rates of organ donation.

Furthermore, since this study revealed a spontaneous decision to donate, it is considered a healthy attitude towards donation. This conclusion is supported by a study in which donors did not feel obliged to make a donation [2, 7–9]. However, since men are more afraid than their counterparts, this can prevent certain donors or let them hesitate to give at any stage. This issue could be addressed through public awareness, predonation psychologists, and the organ donation campaign. Endorsement of liver donation was acknowledged by donors regardless of recipient outcome. Moreover, no donor responded that live liver donation should be abandoned or they felt “forced” to donate, and all donors were satisfied with their donation decision and felt empowered and glad for the donation and saving the life of beloved ones. This is in accordance with the findings of a number of studies [2, 5, 7, 10].

Although our study confirmed that donors were well prepared before and after surgery, the younger age group, particularly males, reported better preparedness, and this may raise concerns about gender difference and level of education. This confirms the same observation that donors are well prepared and educated throughout the donation process [2, 7, 11]. However, as men have pointed out better preparation, it may be necessary to offer an education campaign and sessions through the popular media to educate the general public about organ donation and what they can expect and engage the interdisciplinary advocacy team of donors before the donation process to overcome concerns raised at any time. With regard to pre- and postdonation support, this study found support from the transplant team, family, and friends, and this is similar to the literature that donors feel well supported from their environment [2, 10, 11].

This study revealed an ambiguous feeling before donation which was more prominent among men. As well, it has shown that young people tend to be more anxious and have doubts. These problems would have prevented individuals from making donations. These could be linked to the lack of adequate counselling or psychological support through the donation process. Moreover, these factors might be a normal feeling as a process of uncertainty during the giving and cares of the loved ones. These results are consistent with others [10–12].

### 4.1. Limitation

The present study is limited by the number of participants involved in the survey. As well, donors are surveyed by significant postdonation period, which may limit the level of recall of their experience.

## 5. Conclusions and the Way Forward

We conclude that donors have a positive impact after donating because they are happy to save their lives and be self-reliant by encouraging the giving and saving of lives. Some critical problems have arisen, such as lack of effective pain management, fear and anxiety before surgery, and ambiguous feelings before surgery and fatigue after surgery. These findings underscore the importance of a holistic approach that would enable donors to be well informed prior to surgery. The collaboration of psychologists, pain management, the liver transplant clinic, and social workers can assist in addressing these concerns. In addition, we recommend that future researchers conduct a mixed method study that includes an interview and survey to reveal in depth the experience and challenges and compare the predonation process with the postdonation process. As liver transplantation is a new experiment in Oman, we recommend further investigation to explore public perception of organ donation.

## Figures and Tables

**Table 1:** The demographic characteristics of donors.

	Variable	Frequency (percentage)
Age group	18–27	18 (36%)
	28–38	32 (64%)

Gender	Male	34 (68%)
	Female	16 (32%)

Marital status	Single	20 (40%)
	Married	29 (58%)
	Divorced	1 (2%)

Educational level	Illiterate	1 (2%)
	Elementary	2 (4%)
	Preparatory	1 (2%)
	Secondary	20 (40%)
	High education	26 (52%)

Employment	Student	3 (6%)
	Nonemployee	20 (40%)
	Governmental sector	18 (36%)
	Private sector	7 (14%)
	Free business	2 (4%)

Relation of the donor to the recipient	Parent	30 (60%)
	Sibling	9 (18%)
	Cousin	2 (4%)
	Uncle/aunt	2 (4%)
	Spouse	1 (2%)
	Others	6 (12%)

**Table 2:** Donation process domain in correlation with age and gender.

Question	Response	Total (%)	Age (*P* value)	Gender (*P* value)
How did you hear about liver donation?	Social media/media	6 (12%)	0.334	0.690
	Hospital staff	33 (66%)		
	Relative	9 (18%)		
	Others	2 (4%)		

How much you believe is taken from your liver for donation?	20–30%	13 (26%)	0.006	0.717
	60–65%	28 (56%)		
	50–70%	9 (18%)		

In your opinion, what prevents people from donating organs?	Family objection	4 (8%)	0.036	0.813
	Financial burden	1 (2%)		
	Religious barriers	1 (2%)		
	Lack of knowledge	23 (46%)		
	Health complications	7 (14%)		
	Fear of operation	12 (24%)		
	Others	2 (4%)		

In your opinion, does body image after donation affect the donor decision about donation?	Agree	8 (16%)	0.018	0.032
	Neutral	11 (22%)		
	Disagree	31 (62%)		

Nature of job and workplace affect the donor decision about donation	Agree	22 (44%)	0.133	0.453
	Neutral	5 (10%)		
	Disagree	23 (46%)		

Did you discuss the donation process with family or friends?	Yes	37 (74%)	0.328	0.731
	No	13 (26%)		

Have you felt any pressure to donate?	Yes	3 (6%)	0.99	0.99
	No	45 (90%)		

If yes, from whom?	Recipient	1 (2%)	0.401	0.453
	Family member	2 (4%)		
	Self	2 (4%)		
	None	45 (90%)		

I would recommend people to donate liver to their relative who needs transplantation	Agree	47 (94%)	0.291	0.542
	Neutral	3 (6%)		

I would support organ donation after death	Agree	30 (60%)	0.071	0.961
	Neutral	15 (30%)		
	Disagree	5 (10%)		

**Table 3:** Preparation domain of donors in correlation with age and gender.

Question	Response	Total (%)	Age (*P* value)	Gender (*P* value)
You had sufficient information about donation before starting donation assessment	Agree	37 (74%)	0.247	0.111
	Neutral	10 (20%)		
	Disagree	3 (6%)		

The transplant team prepared me well in terms of education about organ donation	Agree	41 (82%)	0.034	0.246
	Neutral	8 (16%)		
	Disagree	1 (2%)		

The transplant team prepared me well in terms of pain I will encounter after surgery	Agree	40 (80%)	0.087	0.093
	Neutral	5 (10%)		
	Disagree	5 (10%)		

I was informed about recovery after surgery	Agree	38 (76%)	0.520	0.664
	Neutral	6 (12%)		
	Disagree	6 (12%)		

How well do you feel the transplant team prepared you for a potential complication?	Not at all	3 (6%)	0.019	0.748
	Minimally	7 (14%)		
	Somewhat	20		
	Quit a lot	12 (24%)		
	Extremely	8 (16%)		

**Table 4:** Support domain of donors in correlation with age and gender.

Question	Response	Total (%)	Age (*P* value)	Gender (*P* value)
The transplant team and health care providers provided support I need before donation	Agree	41 (82%)	0.253	0.699
	Neutral	9 (18%)		

The transplant team and health care providers provided support I need after donation	Agree	41 (82%)	0.039	9.207
	Neutral	6 (12%)		
	Disagree	3 (6%)		

Family and friends have provided support before donation	Agree	39 (78%)	0.009	0.328
	Neutral	9 (18%)		
	Disagree	2 (4%)		

Family and friends have provided support after donation	Agree	40 (80%)	0.211	0.135
	Neutral	8 (16%)		
	Disagree	2 (4%)		

**Table 5:** Experience domain in correlation with age and gender.

Question	Response	Total (%)	Age (*P* value)	Gender (*P* value)
I feel empowered and satisfied after donation	Agree	45 (90%)	0.004	0.163
	Neutral	5 (10%)		

I feel that my socializing has changed since donation	Agree	14 (28%)	0.384	0.206
	Neutral	9 (18%)		
	Disagree	27 (54%)		

How did this experience compare with your expectations of donation?	Exceeds expectation	17 (34%)	0.016	0.586
	Highly met expectations	12 (24%)		
	Met expectations	15 (30%)		
	Below expectations	2 (4%)		
	Did not meet any expectations	4 (8%)		

How well do you feel the recipient is doing?	Exceeds expectation	23 (46%)	0.004	0.008
	Highly met expectations	14 (28%)		
	Met expectations	11 (22%)		
	Below expectations	2 (4%)		

**Table 6:** Challenges domain in correlation with age and gender.

Question	Response	Total (%)	Age (*P* value)	Gender (*P* value)
I have complete control over donating part of my liver	Agree	48 (96%)	0.99	0.99
	Neutral	2 (4%)		

I felt hesitant to donate at one point before surgery	Agree	8 (16%)	0.571	0.856
	Neutral	7 (14%)		
	Disagree	35 (70%)		

I experienced ambiguous feeling before donation	Agree	14 (28%)	0.223	0.031
	Neutral	8 (16%)		
	Disagree	28 (56%)		

You had fear from being operated on	Agree	15 (30%)	0.103	0.495
	Neutral	11 (22%)		
	Disagree	24 (48%)		

I have experienced anxiety, doubt, or concern	Agree	18 (36%)	0.036	0.836
	Neutral	10 (20%)		
	Disagree	22 (44%)		

## Data Availability

Data used to support the findings of this study may be obtained upon reasonable request by contacting the following authors: Salah Al Awaidy, email Salah.Awaidy@gmail.com, or Mudhar Al Adawi, email mudhar.aladawi@gmail.com.

## References

[1] Song A. T. W., Avelino-Silva V. I., Pecora R. A. A., Vincenzo P., Luiz Augusto C. D’A., Edson A. (2014). Liver transplantation: fifty years of experience. *World Journal of Gastroenterology*.

[2] Schnurman K. K., Zilberfein F., Augurt A., Brosnan M., Song Y. M. (2005). Social work interventions with living related liver donors: the implications for practice. *Progress in Transplantation*.

[3] Lei L., Deng J., Zhang H., Dong H., Luo Y., Luo Y. (2018). Level of organ donation-related knowledge and attitude and willingness toward organ donation among a group of university students in western China. *Transplantation Proceedings*.

[4] Winder G. S., Fontana R. J. (2019). Outcomes in living liver donor “heroes” after the spotlight fades. *Liver Transplantation*.

[5] LaPointe Rudow D., DeLair S., Feeley T. (2019). Longterm impact of living liver donation: a self‐report of the donation experience. *Liver Transplantation*.

[6] Feltrin A., Pegoraro R., Rago C. (2008). Experience of donation and quality of life in living kidney and liver donors. *Transplant International*.

[7] Diaz G. C., Renz J. F., Mudge C. (2002). Donor health assessment after living-donor liver transplantation. *Annals of Surgery*.

[8] Akbulut S., Ozer A., Gokce A., Demyati K., Saritas H., Yilmaz S. (2020). Attitudes, awareness, and knowledge levels of the Turkish adult population toward organ donation: study of a nationwide survey. *World Journal of Clinical Cases*.

[9] Winsett R. P., Russell C., Grewal H. P., Shokouh-Amiri M. H., Gaber A. O. (2003). Perceptions of the donation process from adult-to-adult living liver donors. *Progress in Transplantation*.

[10] Morooka Y., Umeshita K. (2014). Perceptions of transplant surgery among living liver donors in Japan. *Progress in Transplantation*.

[11] Fernandes M. E. N., Bittencourt Z. Z. L. d. C., Boin I. d. F. S. F. (2015). Experiencing organ donation: feelings of relatives after consent. *Revista Latino-Americana de Enfermagem*.

[12] Walter M., Papachristou C., Fliege H. (2002). Psychosocial stress of living donors after living donor liver transplantation. *Transplantation Proceedings*.

